# The implication of cigarette smoking and cessation on macrophage cholesterol efflux in coronary artery disease patients[S]

**DOI:** 10.1194/jlr.P055491

**Published:** 2015-03

**Authors:** Wei Song, Wei Wang, Li-Yang Dou, Yu Wang, Yan Xu, Lian-Feng Chen, Xiao-Wei Yan

**Affiliations:** Peking Union Medical College (PUMC) Hospital, PUMC and Chinese Academy of Medical Sciences, Beijing, China

**Keywords:** macrophages, ABCA1, tobacco

## Abstract

We investigated ATP-binding cassette transporters A1/G1 expression and function in mediating cholesterol efflux by examining the macrophages of cigarette-smoking patients with coronary artery disease (CAD) before and after smoking abstinence. Peripheral blood monocyte cells were collected from nonsmokers (n = 17), non-CAD (NCAD) smokers (n = 35), and CAD smokers (n = 32) before and after 3 months of smoking cessation. We found that the ABCA1 expression level was lower in macrophages from NCAD and CAD smokers than from nonsmokers at baseline. The ABCA1 function of mediating cholesterol efflux was reduced in NCAD and CAD smokers as compared with nonsmokers. After 3 months of smoking cessation, ABCA1 expression and function were improved in CAD smokers. However, ABCG1 expression and function did not change after smoking cessation. Furthermore, ABCA1 expression was inhibited by tar in human acute monocytic leukemia cell line THP-1-derived macrophages through the inhibition of liver X receptors. Nicotine and carbon monoxide did not inhibit ABCA1 expression. Our results indicate that chronic cigarette smoking impaired ABCA1-mediated cholesterol efflux in macrophages and that tobacco abstinence reversed the function and expression of ABCA1, especially in CAD patients. It was tobacco tar, rather than nicotine or carbon monoxide, that played a major role in the tobacco-induced disturbance of cellular cholesterol homeostasis.

Cigarette smoking is an independent risk factor of atherosclerosis (1, 2). In atherosclerotic lesions, the primary cell type is foam cells, which are macrophages overloaded with cholesterol ester. Macrophage overexpression of scavenger receptors, such as CD36, lectin-like oxidized LDL receptor-1, and scavenger receptor A, can internalize modified lipoprotein during differentiation under inflammation and oxidative stress, leading to the overload of cholesterol ester (3, 4). Meanwhile, cholesterol efflux of macrophages can be mediated by different pathways, including ATP-binding cassette transporters, scavenger receptor B1, and aqueous diffusion (5). ATP-binding cassette transporters A1 and G1 (ABCA1/ G1) play pivotal roles and have been shown to have additive activities in cholesterol efflux from macrophages in vivo (6). The imbalance between influx and efflux of cholesterol turns macrophages into lipid-overloaded foam cells.

ABCA1 and ABCG1 are well studied as mediators that regulate cholesterol homeostasis. ABCA1 deficiency was identified as Tangier’s disease, which is characterized by a low level of HDL cholesterol (HDL-C) in patient’s serum (7). Many studies have indicated that ABCA1 is a key player in modulating cholesterol efflux to apolipoprotein A-1 (apoA-1). The dysfunction of this membrane transporter is associated with cellular cholesterol overload and premature atherosclerotic diseases (8–10). Although ABCG1 is ubiquitously expressed in macrophages, data from human and animal models give contradictory evidence regarding whether it is a protector of atherosclerosis (11–13).

Nicotine, tobacco tar, and carbon monoxide (CO) are major alkaloids in cigarette smoke and have been considered, at least partially, responsible for the deleterious effects of tobacco because they are all toxic components of cigarettes. Although nicotine, with the stimulation of the nicotinic acetylcholine receptor α-7 subunit (α-7 nAchR), has now become the focus of attention because of its anti-inflammatory effect (14), other studies have demonstrated that it induces oxidative stress, which may play an important role in the development of atherosclerotic disease (15). CO has been well studied in apoptosis and proliferation (16, 17), and there is little evidence for its association with lipid metabolism.

Although cigarette smoking promotes inflammation, thrombosis, and oxidative stress, the underlying mechanisms involved in the pathophysiology of peripheral blood monocyte cell (PBMC) dysfunction in response to cigarette smoking remain to be understood. Data on which toxic components of tobacco contribute to macrophage cholesterol accumulation are not available. In the present study, we focused on the effects of cigarette smoking and smoking abstinence on ABCA1/G1 expression and function in macrophages from chronic smokers and patients with coronary artery disease (CAD). We also observed the effect of different tobacco components on ABCA1/G1 expression as well as the related signal transduction pathway.

## MATERIALS AND METHODS

**Fig. 1.**
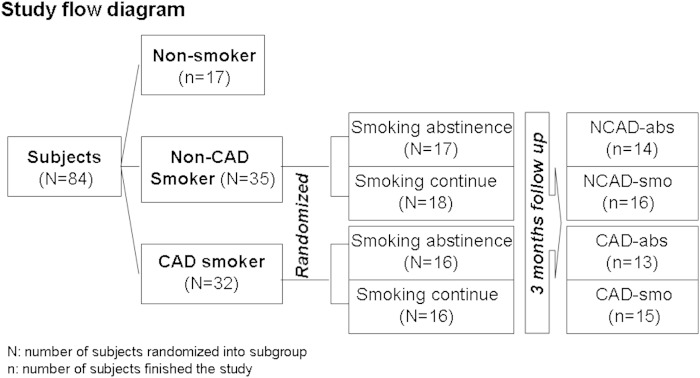
Study flow chart.

This was a randomized, prospective, and parallel controlled study. All the subjects, including 17 nonsmokers, 35 non-CAD (NCAD) smokers, and 32 CAD smokers, were screened at Peking Union Medical College Hospital (PUMCH) (Beijing, China). Smoking subjects eligible for this study were individuals aged 40–80 years who had smoked at least 10 cigarettes per day for 10 years. Smoking subjects were randomized in a 1:1 ratio to either a smoking cessation subgroup or a continued smoking subgroup. In the NCAD smoker group, 17 of 35 smoking subjects were randomized into a smoking abstinence group (NCAD-abs), and 18 subjects comprised a continuing smoking group (NCAD-smo) as a paralleled control. Sixteen of 32 CAD smokers were randomized into a smoking abstinence group (CAD-abs), and 16 were randomized into a smoking group (CAD-smo). Smokers placed into smoking abstinence groups were asked to stop smoking for the 3 month study period. Cotinine concentration in urine and CO of expiration were tested to insure compliance with the study requirements (18). Seventeen nonsmoking subjects were enrolled as a control group (**Fig. 1**).

There were 14 nonCAD smokers and 13 CAD smokers who completed the 90 days of smoking cessation. Four CAD smokers and five nonCAD smokers in smoking cessation groups withdrew during follow-up. All the CAD smokers had documented CAD, including individuals who survived myocardial infarction or experienced angina pectoris and coronary stenosis ≥ 75% in a major epicardial artery by coronary angiography. The exclusion criteria included acute coronary syndrome, diabetes, having taken statin within 2 weeks preceding blood sampling, serum triglyceride ≥ 400 mg/dl, ischemic cerebrovascular or cardiac episodes within 3 months preceding the randomization, and hypertension. Fifty milliliters of blood was collected. All participants showed normal renal function, liver function, and an acceptable lipid profile.

### Ethics statement

The study was registered in the Chinese clinical trial registry (registration number: ChiCTR-RCH-10000748). Ethical approval for the study was obtained from the Human Ethics Committee of PUMCH. Informed consent was obtained from each study subject. The study protocol conformed to the ethical guidelines of the Declaration of Helsinki.

### Reagents

The Ficoll-Paque premium was purchased from Haoyang (Tianjing, China), the human macrophage colony stimulation factor (M-CSF) was from PeproTech, Inc., and 22-[N-nitrobenz-2-oxa-1, 3-diazol-4-yl-amino]23, 24-bisnor-5-cholen-3-ol (NBD-cholesterol) was obtained from Molecular Probes (Eugene, OR). Antibodies against ABCA1, ABCG1, and GAPDH were from Novus Biologicals (ABCAM, Cambridge, MA). The human acute monocytic leukemia cell line THP-1 was obtained from the cell bank of the Basic Research Institute of the Chinese Academy of Medical Sciences (Peking, China). PMA, purified human HDL, and apoA-1 were purchased from Sigma-Aldrich (St. Louis, MO). The fluorescence cholesterol was diluted in ethanol, and the final concentration of ethanol was 0.5%. Cigarette tar, a generous gift from the China National Tobacco Corporation, was dissolved in ethanol. Hydrogen tartrate salt of nicotine, which was also obtained from Sigma, was dissolved in normal saline (0.9% NaCl) to obtain the stock concentration, and the pH of the nicotine solution was adjusted to 7.4 by NaOH (1 M). Tricarbonyldichlororuthenium (II) dimmer (CORM-2)-released CO was used to mimic CO function (17). CORM-2, α- bungarotoxin, and TO901317 were purchased from Sigma.

### Biochemical analysis

Serum biochemical tests, including plasma lipid profile (total cholesterol, TG, LDL-C, HDL-C, apoA-1, and apoB), liver function (alanine aminotransferase, aspartate aminotransferase, bilirubin, albumin/globulin ratio, alkaline phosphatase, and γ-glutamyl transpeptidase), kidney function (Cr and blood urea nitrogen), electrolytes (K, Na, and Cl) (Olympus AU-5400), complete blood count (Sysmex XE-5000-2), and urinalysis (SIEMENS Clinitek Atlas), were conducted in the clinical laboratory of PUMCH.

### Isolation of human monocytes and cell culture

Isolation of PBMCs was performed by gradient centrifugation layering of 50 ml heparinized (10 U/ml) blood. The viability and purity of monocytes were determined by flow cytometric analysis (Accuri Cytometers; BD, Franklin Lakes, NJ) (CD14 staining), confirming that at least 85% purity was achieved. Monocytes were first cultured in a serum-free RPMI 1640 medium containing 25 mmol/l Hepes and 10 ng/ml Human M-CSF for 4–6 h and then cultured in RPMI 1640 containing 10% autologous serum and M-CSF for 7 days at 37°C in humidified incubators with 5% CO_2_. CD68 staining was positive in the PBMC-derived macrophages.

### Analysis of ABCA1 and ABCG1 protein expression

Western blot analysis was performed to analyze ABCA1 and ABCG1 protein expression. Cells were lysed in a lysis buffer containing Tris (50 mmol/l), NaCl (100 mmol/l), NAF (50 mmol/l), EDTA (1 mmol/l), 0.1% SDS, 0.5% deoxycholic acid sodium salt, 1% Triton X-100, and protease inhibitor. Cell lysate was kept on ice for 30 min and then centrifuged at 12,000 *g* for 10 min at 4°C. The protein level was measured using a BCA kit. A total of 80 μg of protein per sample was electrophoresed on 4% to 12% gradient PAGE gel and electroblotted on polyvinylidene difluoride membranes. After blocking the blots for 3 h at room temperature with TBS-T containing 5% BSA and 0.5% Tween 20, membranes were incubated with anti-ABCA1/G1 antibody (1:1,000 dilutions) for 24 h. Immunoreactivity was detected by HRP-conjugated goat anti-mouse secondary antibody. Protein bands were detected with an ECL detection kit (Pierce, Rockford, IL). Densitometry analysis of the immunoreactive band was performed using AlphaEaseFC (Alpha Innotech, Santa Clara, CA).

### Analysis of ABCA1 and ABCG1 mRNA expression

Quantitative real-time PCR was performed to evaluate mRNA expression. RNA was extracted from cells by a TRI reagent (Sigma). Two micrograms of RNA was used for cDNA synthesis with oligo (dT) and SuperScriptIII reverse transcriptase (Invitrogen). For investigation of ABCA1/G1 mRNA, cDNA was synthesized using the Reverse Transcription System for First Strand cDNA Synthesis kit (Promega, Shanghai, China). The thermocycler protocol for the RT phase was one cycle at 20°C for 10 min, one cycle at 42°C for 60 min, and one cycle at 95°C for 5 min. real-time-PCR was performed with specific primers for human ABCA1 (forward: 5′-TACAGCCAGAAAGACACCAG-3′ reverse: 5′-CACA­GTAGACTTTGGGAGAG-3′), ABCG1 (forward: 5′-GCAGTTACT­CTGCAGAGATG-3′ reverse: 5′-CGGAAATTCCTTTCAGGAGG-3′). Human GAPDH primers (forward: 5′-ATGGATGATGATATCGCCGCGC-3′ reverse: 5′-CTAGAAGCATTTGCGGTGGACG-3′) were used for the internal standard. PCR was performed in an ABI 7500 thermocycler (Opticon USA, Renton, WA), and the sample was normalized to GAPDH using the 2^−ΔΔCt^ (cycle threshold) method.

### Cholesterol efflux assay

NBD-cholesterol, a fluorescence analog of cholesterol, was used to evaluate intercellular cholesterol efflux (19, 20). ApoA-1 and HDL were used as the acceptors to evaluate ABCA1- and ABCG1-mediated cholesterol efflux, respectively. Macrophages were incubated in the medium with 5 µM NBD-cholesterol for 4 h, at which time the supernatant was discarded. The cells were washed three times with PBS and then incubated for 4 h with medium containing apoA-1 (50 µg/ml) or HDL (50 µg/ml). The medium was collected and centrifuged at 12,000 *g* for 10 min to remove cell debris, and cells were lysed in 0.1% Triton X-100 for at least 1 h after washing with cold PBS. The fluorescence intensity (FI) of the medium and cell lysate were measured in a black polystyrene 96-well assay plate (Costar; Corning Inc., Corning, NY) using a multilabel counter (PerkinElmer, Waltham, MA) at 469 nm wavelengths for excitation and 537 nm for emission. The calculations were performed as follows: percentage of cholesterol efflux = FI (efflux medium)/FI (efflux medium) + FI (cell lysate), and changes of cholesterol efflux = rate of cholesterol efflux at endpoint − rate of cholesterol efflux at baseline.

### THP-1 culture and treatment

THP-1 monocyte was used to evaluate the effects of different concentrations of nicotine, cigarette tar, and CO on ABCA1 expression in vitro. Cells were cultured in RPMI 1640 medium supplemented with 20% heat-inactivated FBS in 5% CO_2_ at 37°C and were differentiated into macrophages by incubation with 100 ng/ml PMA for 72 h. Cigarette tar stock dissolved in ethanol was diluted by RPMI 1640 to 0.1 g/l. Hydrogen tartrate salt of nicotine was dissolved in basic RPMI 1640 to obtain the required concentrations (ranging from 10^−7^ M to 10 mM); CORM-2 (from 10^−5^ M to 100 mM) was then diluted by RPMI 1640. Macrophages were cultured in the medium containing different concentrations of tar, nicotine, and CO for 72 h. Liver X receptor (LXR) agonist TO901317 was used to detect whether LXR was involved in ABCA1 expression mediated by cigarette tar. Macrophages were preincubated with 1 µM TO901317 for 2 h and then coincubated with cigarette tar for another 72 h. An antagonist of α-7 nAchR, α- bungarotoxin (BTX), was used to investigate whether α-7 nAchR was involved in ABCA1 expression mediated by nicotine. Macrophages were preincubated with 10^−5^ M BTX for 2 h and then stimulated by nicotine for another 72 h. Total protein and mRNA was harvested for ABCA1 detection, and a 3-(4, 5)-dimethylthiahiazo(-z-y1)-3,5-di-phenytetrazoliumromide assay was carried out to evaluate the survival rate of cells.

### Statistical analysis

Data are represented as mean ± SEM. Data were analyzed using the SPSS Statistical Analysis System V-16.0 (SPSS, Chicago, IL). One-way ANOVA and paired *t*-test were used. Wilcoxon signed-rank tests were used for ordinal variables, and *p* < 0.05 was considered to be statistically significant. Data were presented by GraphPad Prism 5 (GraphPad Software, San Diego, CA).

## RESULTS

### Study population

Seventy-five subjects (17 nonsmokers, 30 NCAD smokers, and 28 CAD smokers) completed the study. Subjects who had been randomized into the smoking cessation group and who failed to stop smoking for 90 days, which was assessed by the cotinine in urine and CO in expiration (supplementary Table 1), were excluded from the study.

Baseline plasma apoA-1 and HDL-cholesterol (HDL-C) levels were significantly lower in NCAD smokers (*p* = 0.002 and *p* < 0.001, respectively) and CAD smokers (*p* = 0.019 and *p* = 0.004, respectively) than in nonsmokers. Regarding apoA-1 or HDL-C, there was no statistical disparity between CAD and NCAD smokers. Triglyceride and apoB levels were higher in NCAD smokers than in nonsmokers (*p* = 0.003 and *p* = 0.038, respectively). Other parameters, including age, BMI, blood pressure, plasma TC, LDL-cholesterol, and glucose levels, made no difference among the study groups at baseline (**Table 1**).

**TABLE 1.** Laboratory profile of study population at baseline

Characteristic	Nonsmoker (n = 17)	Smoker		*P* vs. Nonsmoker	
		NCAD (n = 30)	CAD (n = 28)	NCAD	CAD
Age (years)	55.88 ± 12.67	53.37 ± 8.40	57.68 ± 8.12	0.382	0.537
BMI	24.70 ± 2.93	25.81 ± 2.52	25.38 ± 3.26	0.213	0.450
apoA-1 (g/l)	1.58 ± 0.32	1.33 ± 0.20	1.38 ± 0.28	0.002*^a^*	0.019*^a^*
apoB (g/l)	0.88 ± 0.14	0.99 ± 0.19	0.81 ± 0.16	0.038*^a^*	0.568
TC (mmol/l)	4.99 ± 0.81	5.09 ± 0.81	4.52 ± 0.77	0.645	0. 058
TG (mmol/l)	1.04 ± 0.44	2.20 ± 1.44	1.74 ± 1.35	0.003*^a^*	0.071
HDL-C (mmol/l)	1.41 ± 0.30	1.12 ± 0.22	1.17 ± 0.29	<0.001*^a^*	0.004*^a^*
LDL-C (mmol/l)	3.06 ± 0.53	3.15 ± 0.85	2.66 ± 0.76	0.696	0.086
Glucose (mmol/l)	4.90 ± 0.61	6.01 ± 3.81	5.86 ± 0.13	0.144	0.231
SBP (mm Hg)	118 ± 12	122 ± 8	124 ± 14	0.217	0.131
DBP (mm Hg)	74 ± 10	72 ± 8	72 ± 12	0.508	0.583

All data normally distributed variables are expressed as mean and standard deviation. CAD, coronary artery disease; DBP, diastolic blood pressure; HDL-C, high-density lipoprotein cholesterol; LDL-C, low-density lipoprotein cholesterol; SBP, systolic blood pressure; TC, total cholesterol.

a *P* < 0.05 obtained in the comparison between CAD or healthy smokers and nonsmokers using ANOVA.

After 3 months of smoking cessation, plasma apoA-1 levels increased more significantly in CAD-abs (0.32 ± 0.30 g/l) than in CAD-smo (0.02 ± 0.29 g/l; *p* = 0.032) but did not change in NCAD-abs. The change of plasma HDL-C levels in CAD-abs (0.04 ± 0.18 mmol/l) was comparable to the change in CAD-smo (0.13 ± 0.13 mmol/l) (*p* = 0.203). There were no significant differences in other biochemical parameters between the study groups (**Table 2**).

**TABLE 2.** Laboratory profile of study population after 3 month follow-up

Characteristic	NCAD-smo (n = 16)	NCAD-abs (n = 14)	*P*	CAD-smo (n = 15)	CAD-abs (n = 13)	*P*
apoA-1 (g/l)	0.09 ± 0.10	0.14 ± 0.25	0.486	0.02 ± 0.29	0.32 ± 0.34	0.032*^a^*
apoB (g/l)	−0.01 ± 0.09	−0.03 ± 0.33	0.891	−0.02 ± 0.26	−0.03 ± 0.23	0.913
TC (mmol/l)	−0.10 ± 0.36	−0.14 ± 0.79	0.867	−0.05 ± 0.17	−0.26 ± 0.38	0.343
TG (mmol/l)	−0.07 ± 0.47	−0.05 ± 0.36	0.925	−0.12 ± 0.35	−0.09 ± 0.47	0.882
HDL-C (mmol/l)	0.10 ± 0.09	0.20 ± 0.26	0.184	0.04 ± 0.18	0.13 ± 0.13	0.203
LDL-C (mmol/l)	−0.10 ± 0.46	−0.14 ± 0.44	0.839	−0.04 ± 0.64	−0.21 ± 0.45	0.578

CAD-abs, coronary artery disease abstinence group; CAD-smo, coronary artery disease smoking group; HDL-C, high-density lipoprotein cholesterol; LDL-C, low-density lipoprotein cholesterol; NCAD-abs, non-coronary artery disease abstinence group; NCAB-smo, non-coronary artery disease smoking group; TC, total cholesterol.

a *P* < 0.05 obtained in the comparison between cessation and continue using independent-samples *t*-test.

### ABCA1 expression and its function among study groups at baseline

**Fig. 2.**
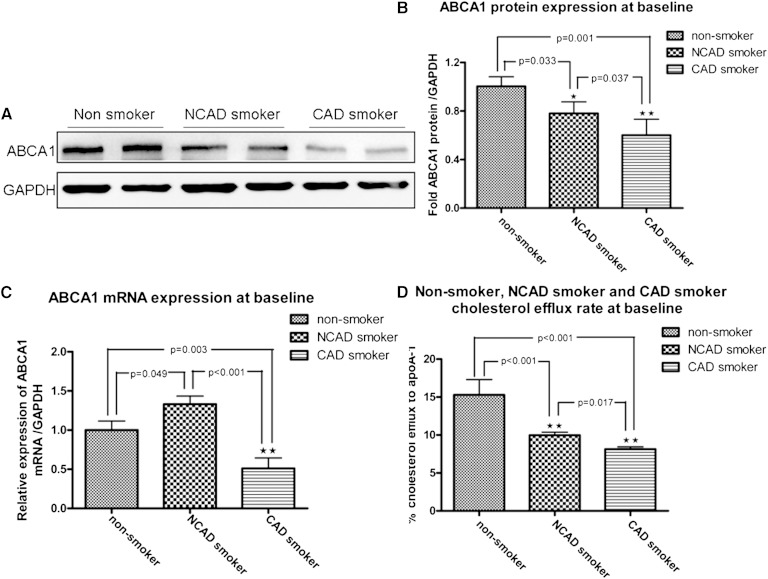
ABCA1 expression and its function among study groups at baseline. A: ABCA1 protein expression was suppressed in macrophages from NCAD and CAD smokers compared with those from nonsmokers (each group, n = 6). B: Densitometry (*p* = 0.033, *p* < 0.001 vs. nonsmoker). C: ABCA1 mRNA was dramatically suppressed in CAD smokers but was upregulated in macrophages from NCAD smokers compared with that from nonsmokers (*p* < 0.001; each group, n = 10). D: ApoA-1-mediated cholesterol efflux was decreased in macrophages from CAD smokers (*p* < 0.001; n = 26) and NCAD smokers (*p* < 0.001; n = 24) compared with those from nonsmokers (n = 10) at baseline. ApoA-1-mediated cholesterol efflux was significantly lower from CAD smokers than from NCAD smokers (*p* = 0.017).

At baseline, ABCA1 protein expression was significantly lower in macrophages from CAD smokers and NCAD smokers, as compared with the expression from nonsmokers (*p* = 0.001 and *p* = 0.033, respectively). Compared with NCAD smokers, ABCA1 expression was further inhibited in macrophages from CAD smokers (*p* = 0.037) (**Fig. 2A, B**). ABCA1 mRNA expression in macrophages from CAD smokers was significantly reduced compared with nonsmokers (*p* < 0.001), whereas the expression was augmented in macrophages from NCAD smokers compared with that from nonsmokers (*p* = 0.049) (Fig. 2C). There was no difference in ABCG1 expression among the study groups (supplementary Fig. 1).

ABCA1-mediated cholesterol efflux to apoA-1 was attenuated in macrophages from CAD smokers (8.15 ± 1.50%) compared with nonsmokers (15.30 ± 5.70%; *p* < 0.001). It was also diminished in NCAD smokers (9.97 ± 2.00%) compared with nonsmokers (15.30 ± 5.70%; *p* < 0.001). ABCA1-mediated cholesterol efflux in CAD smokers was also lower than in NCAD smokers (*p* = 0.017) (Fig. 1D).

### Changes of ABCA1 expression and function in macrophages after smoking cessation from CAD smokers

**Fig. 3.**
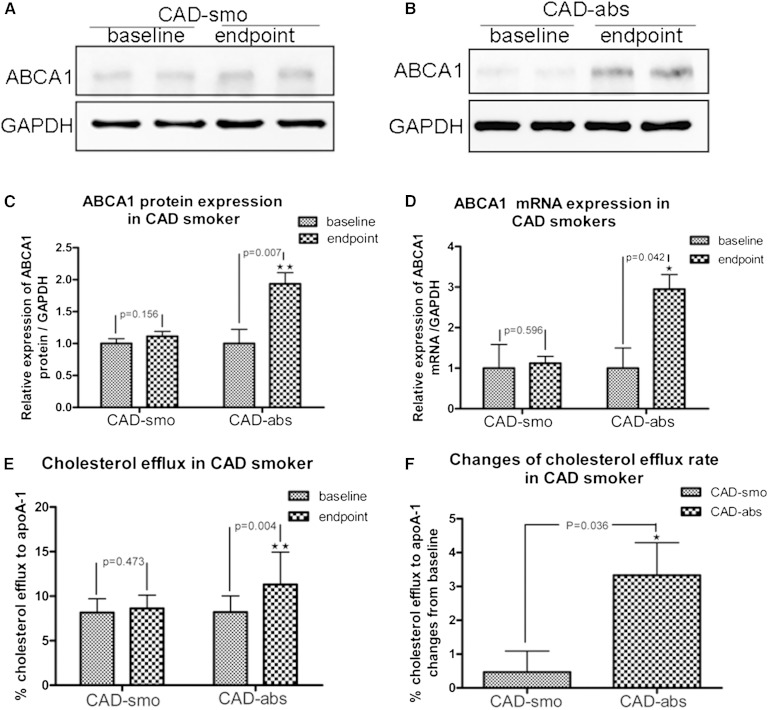
Changes of ABCA1 expression and function in macrophages after smoking cessation from CAD smokers. ABCA1 protein expression did not changed in CAD-smo (*p* = 0.156; n = 6) (A and C), but it was increased after smoking cessation for 3 months in CAD-abs (*p* = 0.007; n = 6) (B and 3). D: ABCA1 mRNA expression was upregulated in macrophages from CAD-abs (*p* = 0.042; n = 10). E: After 3 months of smoking cessation, ABCA1-mediated cholesterol efflux was increased significantly compared with baseline in CAD-abs (*p* = 0.004; n = 12), whereas there was no change in CAD-smo (*p* = 0.473; n = 10). F: After 3 months, ABCA1-mediated cholesterol efflux increased significantly in CAD-abs compared with that in CAD-smo (*p* = 0.036).

Protein and mRNA expression of ABCA1 in macrophages from CAD-abs subjects were increased after 3 months of smoking cessation (**Fig. 3A, C, D**) (all with significance levels of *p* < 0.05 compared with baseline). There was no significant change in ABCA1 expression in macrophages from CAD-smo subjects (Fig. 3B–D). Compared with baseline, at the end of 3 months, ABCA1-mediated cholesterol efflux to apoA-1 was significantly increased in CAD-abs (8.14 ± 1.61% vs. 11.47 ± 3.61%; *p* = 0.004) but not in CAD-smo (8.16 ± 1.55% vs. 8.62 ± 1.49%; *p* = 0.473) (Fig. 3E). The changes of ABCA1-mediated cholesterol efflux between CAD-abs and CAD-smo after a 3 month follow-up were still significantly different (3.33 ± 3.72% vs. 0.64 ± 1.97%; *p* = 0.036) (Fig. 3F).

ABCG1 mRNA expression was upregulated dramatically (*p* < 0.001) in macrophages from CAD-abs after 3 months of tobacco abstinence, whereas ABCG1 protein expression was not increased and the function was not improved (supplementary Fig. 2).

### Changes of ABCA1 expression and function in macrophages from NCAD smokers after smoking cessation

**Fig. 4.**
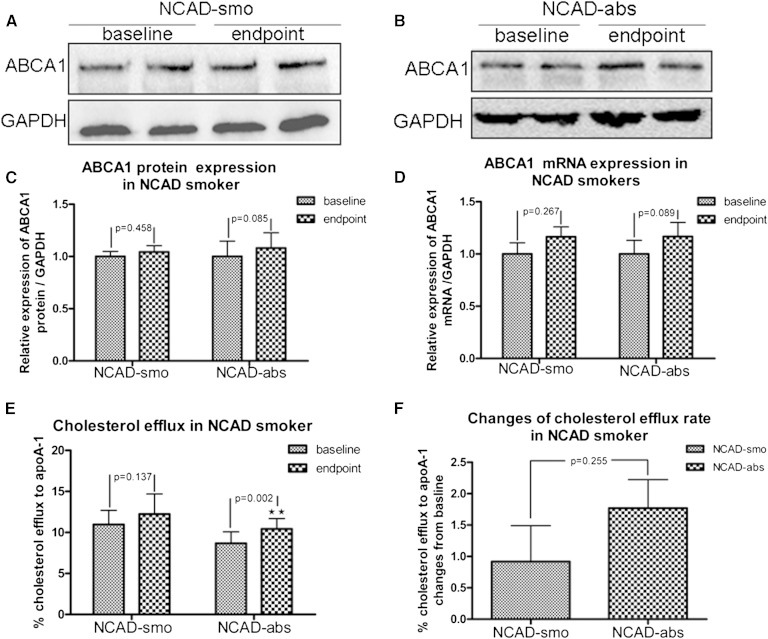
Changes of ABCA1 expression and function in macrophages from NCAD smokers after smoking cessation. A–C: ABCA1 protein expression did not change in macrophages from NCAD-smo and NCAD-abs (*p* = 0.458; n = 6). D: ABCA1 mRNA expression did not change in NCAD-smo and NCAD-abs (*p* = 0.267 and 0.089; n = 10). E: After 3 months of smoking cessation, ABCA1-mediated cholesterol efflux was increased significantly in NCAD-abs (*p* = 0.002; n = 12), whereas there was no change in NCAD-smo (*p* = 0.137; n = 12). F: The changes in ABCA1-mediated cholesterol efflux in NCAD-abs were not significantly different from those in NCAD-smo (*p* = 0.255).

There was no change in ABCA1 protein and mRNA expression in NCAD-abs (**Fig. 4A, C**) and NCAD-smo (Fig. 4B–D). Compared with baseline, ABCA1-mediated cholesterol efflux to apoA-1 was improved in macrophages from NCAD-abs (9.33 ± 2.01% vs. 11.10 ± 1.96%; *p* = 0.002) but not from NCAD-smo (10.61 ± 1.84% vs. 11.53 ± 2.78%; *p* = 0.137) after follow-up at 3 months (Fig. 4E). However, the absolute changes of cholesterol efflux did not reach statistical significance between the two study groups (1.77 ± 1.57% vs. 0.92 ± 1.98; *p* = 0.255) (Fig. 4F). There appeared to be no differences in ABCG1 expression and function from NCAD smokers after tobacco abstinence (supplementary Fig. 3).

### Inhibition of ABCA1 expression from cigarette tar in THP-1-derived macrophages

**Fig. 5.**
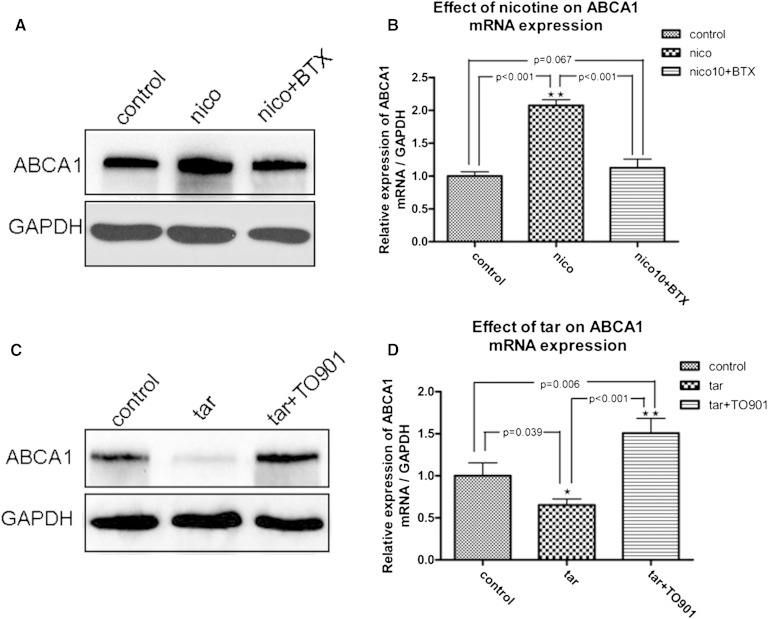
Inhibition of ABCA1 expression from cigarette tar in THP-1-derived macrophages. THP-1 cells were differentiated into macrophages. Macrophages were incubated in medium containing tar or nicotine for 72 h. A: ABCA1 protein expression was stimulated by 100 ng/ml nicotine, which could be reversed by BTX. C: ABCA1 protein expression was inhibited by 0.1 g/l tar, which could be abolished by LXR agonist TO901317. B and D: ABCA1 mRNA was upregulated by nicotine (*p* = 0.006 vs. control) and downregulated by tar (*p* = 0.039 vs. control). The effects of nicotine and tar on ABCA1 mRAN expression could be removed by BTX (*p* < 0.001 vs. nicotine) and TO901317 (*p* < 0.001 vs. tar), respectively.

ABCA1 protein and mRNA expression was enhanced by incubation with 100 ng/ml nicotine (**Fig. 5A, B**) but was inhibited by 0.1 g/l tar (Fig. 5C, D). The LXR agonist TO901317 abolished the inhibitive effect of tar on protein and mRNA expression of ABCA1 (Fig. 4C, D). BTX, a selective α-7-n acetylcholine receptor (α-7-nAchR) antagonist, inhibited the upregulation of ABCA1 induced by nicotine (Fig. 5A, B). The 3-(4, 5)-dimethylthiahiazo(-z-y1)-3,5-di-phenytetrazoliumromide assay was carried out to evaluate the toxic reaction and survival rate of cultured cells stimulated by these agents (supplementary Fig. 4). The survival rate of cells was not affected by stimulation by tar and nicotine. ABCA1 expression was not affected by the administration of CORM-2 (supplementary Fig. 5).

## DISCUSSION

The evidence linking cigarette smoking with cardiovascular disease are shown in many studies; however, the mechanisms involved in cigarette exposure associated cardiovascular dysfunction have been largely debated. Previous studies have proven that tobacco aggravates inflammation, thrombosis, oxidation of LDL, and oxidative stress reaction (21–23). Our study was focused on whether tobacco affects cellular cholesterol metabolism from the lipid metabolic point of view. We found that ABCA1 protein expression and the function of mediated intercellular cholesterol efflux were deficient in CAD smokers. This deficiency might play a detrimental role in cellular cholesterol metabolism and lead to intercellular cholesterol disposal, which may accelerate the formation of foam cells. Many studies have proven that defects in ABCA1 impaired apoA-1-mediated intercellular lipid efflux in early atherosclerosis (8, 24, 25). Apart from its role in lipid metabolism, ABCA1 has also been implicated in engulfing apoptotic cells, inhibiting the release of inflammatory mediators, and binding to other lipoproteins (including apolipoprotein E), thus playing various roles in its antiatherogenic effect (26, 27). Deficiency in ABCA1 protein expression in smokers may attenuate the role of anti-inflammation and antioxidation, resulting in atherosclerotic aggravation.

Regarding the results of this study, it was interesting that ABCA1 mRNA was upregulated in NCAD smokers, which is inconsistent with the results from ABCA1 proteins. Sticozzi et al. (28) also found that ABCA1 mRNA expression was upregulated in keratinocyte exposure to air filled with cigarette smoking. However, as opposed to our study, the conclusion reached by Sticozzi et al. (28) was based on cultured cell lines’ in vitro exposure for only 50 min; there was 92% gas, which was not hydrosoluble, and 8% particulates in the air filled with cigarette smoke, including nicotine and tar (29). Thus, the study by Sticozzi et al. (28) was a more passive smoking model, whereas our data from subjects who had been active long-time smokers were a direct assessment of the exposure-response relationship between cigarette smoking and macrophage ABCA1 expression and function.

The plasma HDL-C level was inversely correlated with the risk of clinical events resulting from atherosclerosis. In our study, the HDL-C level was significantly low in all smokers, which is compatible with other observations that smokers have a low concentration of plasma HDL-C (−5.7%) and apoA-1 (−4.2%) (30). The mechanisms involved in diminishing HDL-C have not yet been clarified. We found that plasma apoA-1 was also reduced in all smokers. ABCA1-dependent lipid transfer to apoA-1 is the rate-limiting step in the biogenesis of nascent HDL, and it also plays a key role in the cardioprotective function of HDL (31). It has been proven that removing ABCA1 in macrophages does not affect the circulation of HDL cholesterol (32). ABCA1 in the liver and intestines is essential for maintaining plasma HDL cholesterol levels (33). Thus, the deficiency of ABCA1 protein expression and dysfunction in macrophages probably reflect what is occurring in liver ABCA1 expression, which results in low plasma HDL cholesterol in smokers.

Moreover, after 3 months of smoking cessation, ABCA1-mediated cholesterol efflux to apoA-1 was dramatically elevated and was accompanied by upregulation of ABCA1 protein expression in macrophages from CAD patients. Although increased plasma HDL-C levels did not reach statistical significance, apoA-1 improvement was significant in the CAD smoking cessation subgroup. This fact reemphasizes the importance of the therapeutic strategy for CAD smokers in quitting tobacco in addition to standard pharmacotherapy treatments.

Why is ABCA1 function reversed in subjects with CAD but not in NCAD subjects? We found that PPAR-γ mRNA was upregulated, whereas ABCA1 was decreased, in CAD smokers compared with NCAD smokers at baseline (supplementary Fig. 6A; all *p* < 0.05). PPAR-γ protein expression was obviously more enhanced in macrophages from CAD than from NCAD smokers (supplementary Figs. 6B, C; *p* < 0.01). These results imply that the mechanism of ABCA1 reversion through smoking cessation may be different for CAD and NCAD smokers. Furthermore, we extended the smoking cessation time to 5 months (post-endpoint) and found that ABCA1 mRNA (supplementary Fig. 7A) and protein expression (supplementary Fig. 7B, C) were enhanced compared with baseline in macrophages from NCAD smokers (both *p* < 0.001). Changes in the ABCA1-mediated cholesterol efflux rate was increased significantly (*p* = 0.005), but there was no change in PPAR-γ expression (supplementary Fig. 7D). We are not sure whether the activation of PPAR-γ is the reason for CAD or a compensatory reaction of CAD. However, Mehrabi et al. (34) found that the expression of PPAR-γ differed according to organ. Thus, it is reasonable to suggest that PPAR-γ in macrophages is increased in certain pathological conditions, such as CAD. Although the role of PPAR-γ in the development of heart disease is controversial, the activation of PPAR-γ/LXR-α-induced ABCA1 transcription supports the hypothesis that is has an effect on its development (35, 36). It may be that activation of PPAR-γ leads to a fast reversal of ABCA1 expression and function.

ABCG1-mediated intercellular cholesterol efflux to HDL was shown to be coordinated with ABCA1 in removing excess cellular cholesterol, and it also affected inflammatory cellular cytokine secretion by modulating cholesterol content in the plasma membrane and within intracellular compartments (33). We did not find any significant difference in ABCG1 expression and function among these groups before or after smoking cessation, which suggests that ABCG1 might not be critical in tobacco-induced changes of macrophage cholesterol metabolism.

Because of the complexity of cigarettes, it is difficult to identify the components responsible for pathophysiologic actions and discern the relevant mechanisms. To understand the effects of tobacco ingredients on macrophage ABCA1 expression, we designed in vitro experiments with THP-1 cells. We found that cigarette tar inhibited ABCA1 expression in THP-1 macrophages, whereas the LXR agonist could abolish this inhibitive effect. This may explain why cigarette smoking reduces ABCA1 expression and function.

Studies in the last century have demonstrated that cigarette tar leads to DNA damage (37, 38). Recent research demonstrates that accumulation of tar-like substances in human neutrophils leads to atypical cell death, which shares features of apoptosis, autophagy, and necrosis (39). We found for the first time that tobacco tar inhibits ABCA 1 expression, which may lead to impairment of cholesterol-mediated efflux in macrophages. Nicotine is probably the most frequently studied component of cigarette smoke, and the plasma levels of nicotine range from 10 ng/ml to 40 ng/ml after exposure to cigarette smoke (40). In our study, we found that nicotine, which was greater than or equal to the plasma nicotine concentration, increased the ABCA1 mRNA and protein expression in THP-1-derived macrophages. Nicotine may responsible for the results from Sticozzi et al. (28), who stimulated keratinocytes with cigarette smoke and found upregulation of ABCA1 expression. Although high doses of nicotine favor atherogenic changes in various models, the majority of evidence suggests that nicotine, at concentrations similar to a smoker’s blood level, has a minor effect on the initiation or propagation of atherosclerosis (41, 42). Some studies even show that nicotine plays an anti-inflammatory role that is mediated by the cholinergic pathway (14). CO is one of the more toxic agents in incompletely burned tobacco. There is sufficient evidence to suggest that CO affects the cardiovascular, respiratory, and central nervous systems (43, 44). In this study, however, CO did not interrupt the ABCA1 expression in THP-1 macrophages. There are thousands of components in cigarettes, and an abundance of work remains to be performed in exploring which components are responsible for the impaired cholesterol metabolism in macrophages. It was more effective to perform this study in vivo than in vitro.

Our study investigated the relationships between cigarette smoking, ABCA1/G1 expression, and the function of cholesterol efflux in macrophages from humans. Cigarette smoking is associated with impaired intercellular cholesterol efflux resulting from the inhibition of ABCA1 expression and function, which is also associated with the reduction of plasma HDL-C levels. Tobacco tar, as a complex in tobacco, plays an important role in inhibiting ABCA1 expression. Our findings provide novel evidence that smoking cessation is an effective method for restoring ABCA1-mediated cholesterol efflux and raising HDL-C levels in CAD patients.

## Supplementary Material

Supplemental Data
