# Supplementary material for: The implication of cigarette smoking and cessation on macrophage cholesterol efflux in coronary artery disease patients
Source: J Lipid Res. 2015 Mar;56(3):682–91. doi: 10.1194/jlr.P055491 (PMC4340315; doi:10.1194/jlr.P055491)
Supplement: Supplemental Data [file supp_56_3_682__index.html]

The Implication of Cigarette Smoking and Cessation on Macrophage Cholesterol Efflux in Coronary Artery Disease Patients — The implication of cigarette smoking and cessation on macrophage cholesterol efflux in coronary artery disease patients — Supplemental Data 

# The implication of cigarette smoking and cessation on macrophage cholesterol efflux in coronary artery disease patients

## Supplemental Data

**Files in this Data Supplement:**

- Supplemental table 1 or fig 1 - Effect of smoking cessation on ABCG1 expression
