# Supplementary material for: The implication of cigarette smoking and cessation on macrophage cholesterol efflux in coronary artery disease patients
Source: J Lipid Res. 2015 Mar;56(3):682–91. doi: 10.1194/jlr.P055491 (PMC4340315; doi:10.1194/jlr.P055491)
Supplement: Supplemental Data [file supp_P055491_jlr.P055491-1.pdf]

Supplementary Figure.

Supplementary Figure 1： ABCG1 expression in macrophages from subjects at the baseline. 1A and 1B showed there was no obvious difference on ABCG1 protein expression in macrophages from three subgroups (each group n=6). 1C showed ABCG1 mRNA expression was no significant disparity in macrophages from NCAD smokers (n=14), CAD smokers (n=13) compared with non-smoke subjects (n=10). Macrophages, from non-smokers (n=10), NCAD smokers (n=22) and CAD smokers (n=20), were used to evaluate cholesterol efflux rate presented in 1D. ABCG1 mediated cholesterol efflux appeared to be a decreased tendency in macrophages from CAD smokers, but it did not reach a statistical difference (p=0.060).

Supplementary Figure 2： Changes of ABCG1 expression and function after smoking cessation in macrophages from CAD subjects. ABCG1 protein expression was no change in both CAD-smo group and CAD-abs group after three months follow-up (each group n=6) (2A, 2B and 2C). 2D showed ABCG1 mRNA expression was up-regulated in macrophages from CAD-abs subjects (each group n=7). 2E showed that ABCG1 mediated cholesterol efflux was not changed in macrophages from CAD-abs group (2.89%±7.36, p=0.221, n=12) or from CAD-smo group (-1.60%±3.27, p=0.136, n=10). 2F showed there was no significant increased in ABCG1 mediated cholesterol efflux in CAD-abs group (p=0.079).

Supplementary Figure 3： ABCG1 expression in macrophages from NCAD smoker subjects after three months smoking cessation. ABCG1 protein expression changed in macrophages from neither NCAD-abs group nor NCAD-smo group (each group n=6) (3A, 3B and 3C). 3D

showed ABCG1 mRNA expression was consistent with protein expression (each group n=10). 3E showed that ABCG1 mediated cholesterol efflux was not obviously improved in macrophages from NCAD-abs (1.66%±3.03, p=0.140, n=10) or from NCAD-smo group (-0.28%±2.40, p=0.724, n=10). 3F showed changes of cholesterol efflux seem to be no dramatically improved (p=0.140).

Supplementary Figure 4: MTT assay. THP-1 macrophages were incubated with 5 mg/ml MTT solution for 4 hours after the stimulation of tar or nicotine. OD value (570nm-630nm) presented the cells survival rate. There was no difference between 0.1 g/L tar and 10^{-5}M nicotine compared with control at different time spots (all p>0.05 vs. control).

Supplementary Figure 5: Effect of CO released by CORM on ABCA1 expression in THP-1 derived macrophages. THP-1 cells were differentiated into macrophages by PMA, and then cells were incubated with different concentration of CO released by CORM. Neither low concentration of CO nor high concentration of CO affected ABCA1 protein expression compared with control.

Supplementary Figure 6: Comparison of ABCA1 and PPAR-γ expression in macrophages from NCAD smoker and CAD smoker at baseline. 6A showed ABCA1 mRNA was dramatically suppressed in CAD smoker compared with non-smoker and NCAD smoker (p=0.01 and p<0.01), but PPAR-γ was obviously up-regulated in macrophages from CAD smoker compared with that from non-smoker and NCAD smoker (p<0.01). ABCA1 and PPAR-γ mRNA expression was no difference between non-smoker and NCAD smoker (p=0.21 and p=0.94). 6B and 6C showed ABCA1 protein expression was suppressed in

macrophages from CAD smoker and NCAD smoker compared with that from non-smoker ($p<0.01$ and $p=0.01$), there was further decreased in CAD smoker compared to NCAD smoker ($p=0.03$), but PPAR-γ protein expression was enhanced in CAD compared with non-smoker and NCAD smoker (both $p<0.01$) (n=8).

Supplementary Figure 7: ABCA1 and PPAR-γ expression after 5 months smoking cessation (post-endpoint). 7A showed ABCA1 mRNA expression was up-regulated at post-endpoint compared to baseline ($p<0.001$), but PPAR-γ mRNA expression was not different ($p=0.225$). 7B. ABCA1 was increased in macrophages from NCAD subjects at post-endpoint compared with that at baseline ($p<0.001$), but there was no change in PPAR-γ protein expression ($p=0.238$). Densitometry was shown as in 7C. 7D showed that after five months smoking cessation, changes of ABCA1 mediated cholesterol efflux was increased significantly ($p=0.005$) (n=10).

Supplementary table Ⅰ: Cotinine in urine and Carbon oxide in expiration

Characteristic		NCAD smokers Baseline	NCAD smokers Endpoint	P vs. Base	CAD smokers Baseline	CAD smokers Endpoint	P vs. Base
Cotinine	smo	1997.6	1789.5	—	1786.5	1902.3	—
	abs	2019.7	54.0	—	2077.5	31.8	—
CO	smo	4.50±0.97	4.29±1.20	0.637	3.82±0.87	4.46±0.78	0.093
	abs	4.43±1.09	2.43±0.85	0.000	4±1.18	2.08±0.64	0.001

P value was obtained in comparison between endpoint and baseline using Wilcoxon signed rank test (Baseline, Base).

Supplementary Figure

Figure 1A

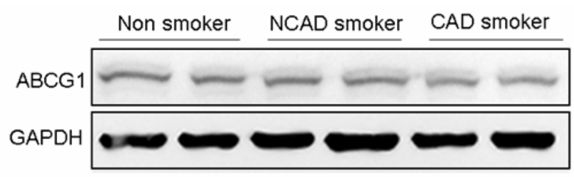

Figure 1B

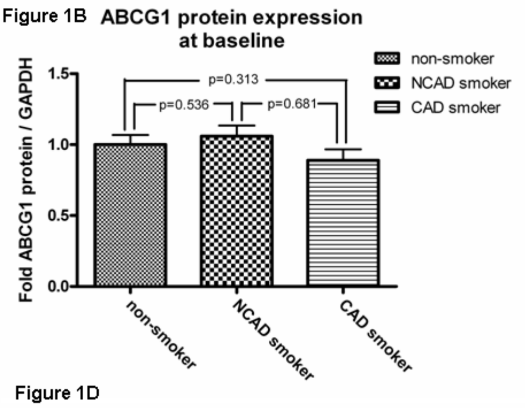

Figure 1C

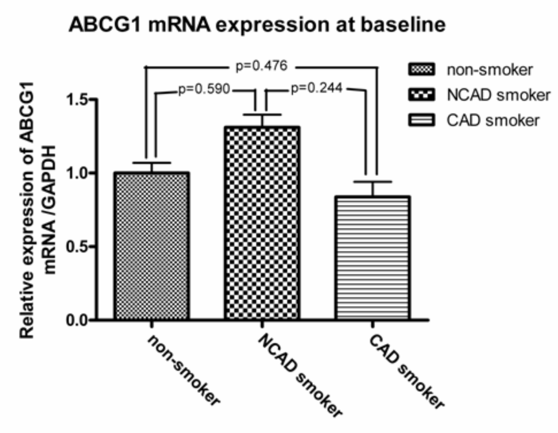

Figure 1D

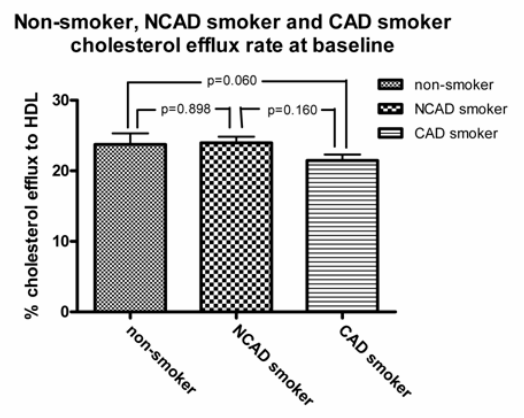

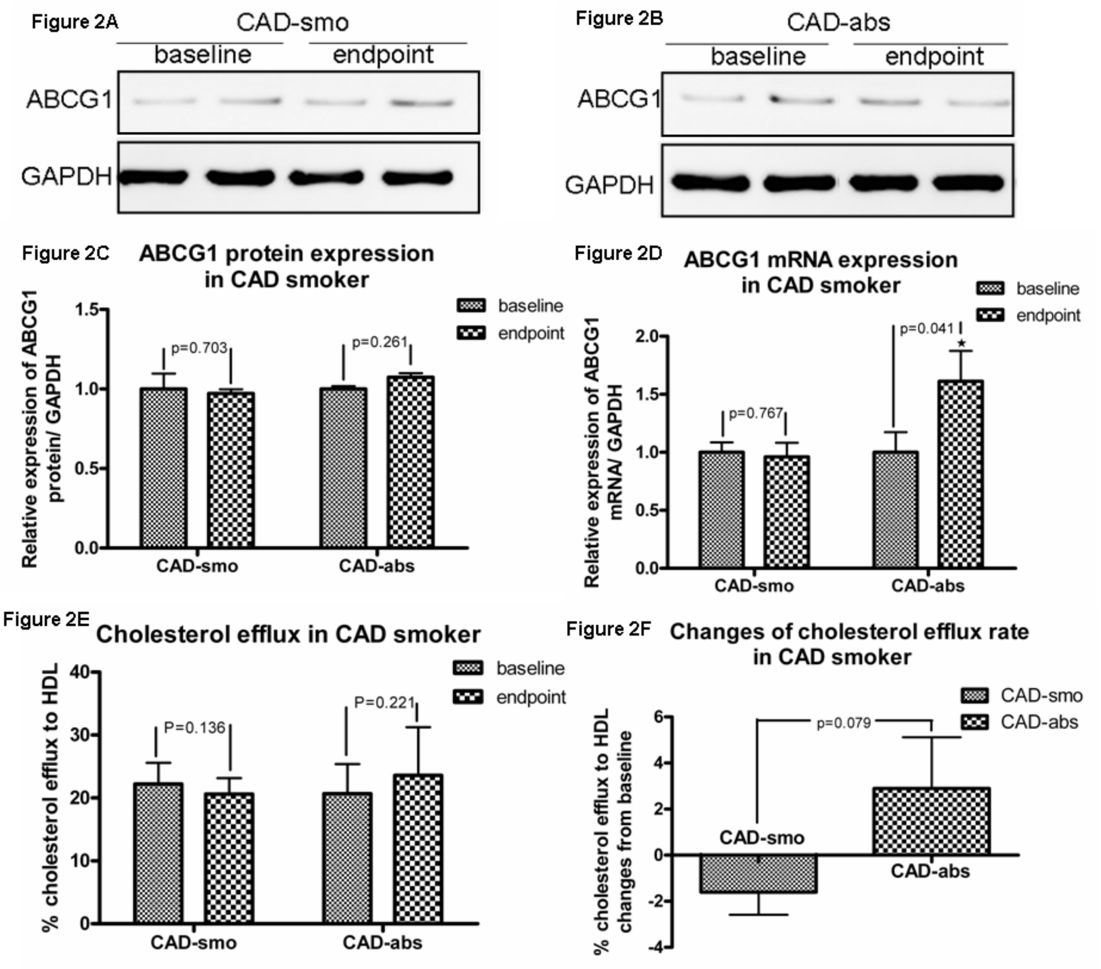
Figure 2A
CAD-smo
baseline
endpoint
ABCG1
GAPDH
Figure 2B
CAD-abs
baseline
endpoint
ABCG1
GAPDH
Figure 2C
ABCG1 protein expression in CAD smoker
Relative expression of ABCG1 protein/ GAPDH
baseline
endpoint
p=0.703
p=0.261
CAD-smo
CAD-abs
Figure 2D
ABCG1 mRNA expression in CAD smoker
Relative expression of ABCG1 mRNA/ GAPDH
baseline
endpoint
p=0.767
p=0.041
*
CAD-smo
CAD-abs
Figure 2E
Cholesterol efflux in CAD smoker
% cholesterol efflux to HDL
baseline
endpoint
P=0.136
P=0.221
CAD-smo
CAD-abs
Figure 2F
Changes of cholesterol efflux rate in CAD smoker
% cholesterol efflux to HDL changes from baseline
CAD-smo
CAD-abs
p=0.079

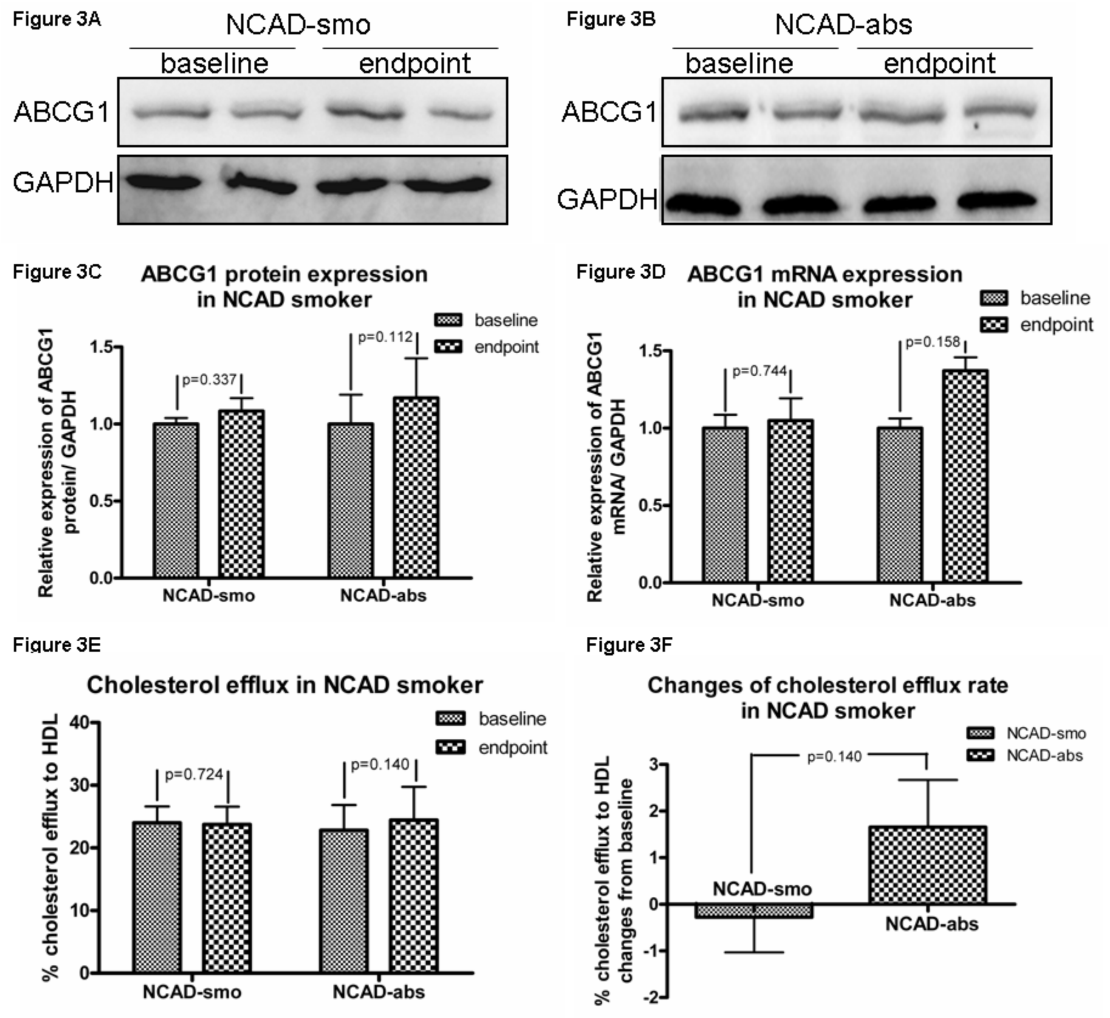
Figure 3A
NCAD-smo
baseline
endpoint
ABCG1
GAPDH
Figure 3B
NCAD-abs
baseline
endpoint
ABCG1
GAPDH
Figure 3C
ABCG1 protein expression in NCAD smoker
baseline
endpoint
Relative expression of ABCG1 protein/ GAPDH
p=0.337
p=0.112
NCAD-smo
NCAD-abs
Figure 3D
ABCG1 mRNA expression in NCAD smoker
baseline
endpoint
Relative expression of ABCG1 mRNA/ GAPDH
p=0.744
p=0.158
NCAD-smo
NCAD-abs
Figure 3E
Cholesterol efflux in NCAD smoker
baseline
endpoint
% cholesterol efflux to HDL
p=0.724
p=0.140
NCAD-smo
NCAD-abs
Figure 3F
Changes of cholesterol efflux rate in NCAD smoker
NCAD-smo
NCAD-abs
% cholesterol efflux to HDL changes from baseline
p=0.140

Figure.4

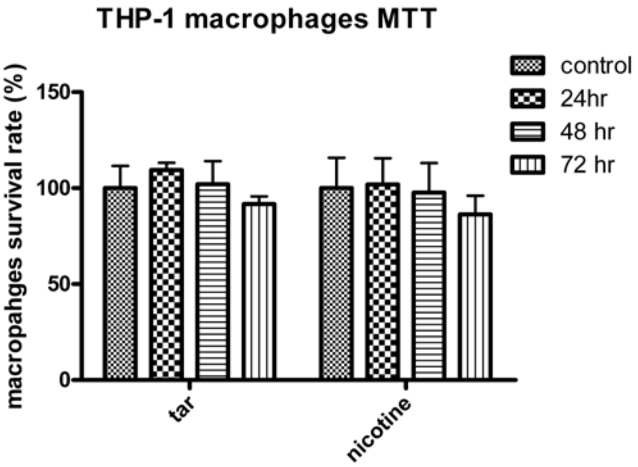
THP-1 macrophages MTT
macropahges survival rate (%)
150
100
50
0
tar
nicotine
control
24hr
48 hr
72 hr

Figure.5

Effect of different concentration of CORM released CO on THP-1 macrophages

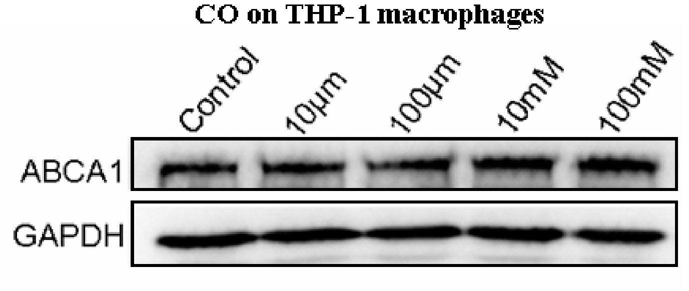

Figure 6A

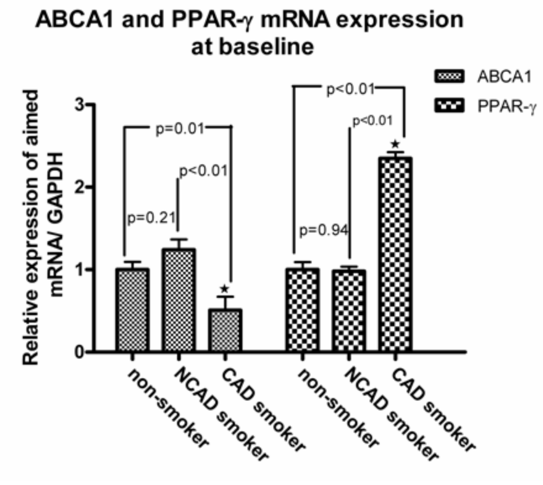

Figure 6B

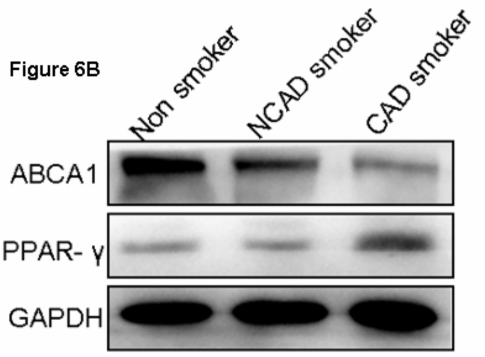

Figure 6C ABCA1 and PPAR-γ protein expression at baseline

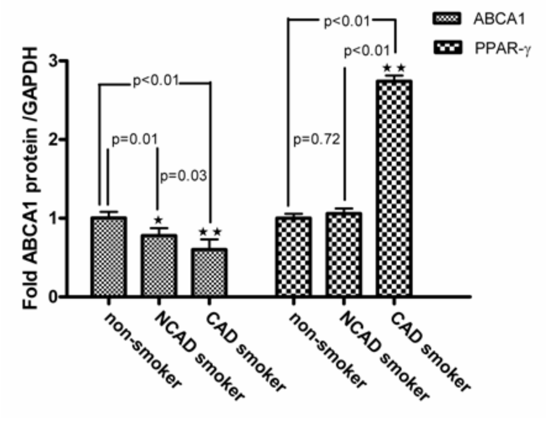

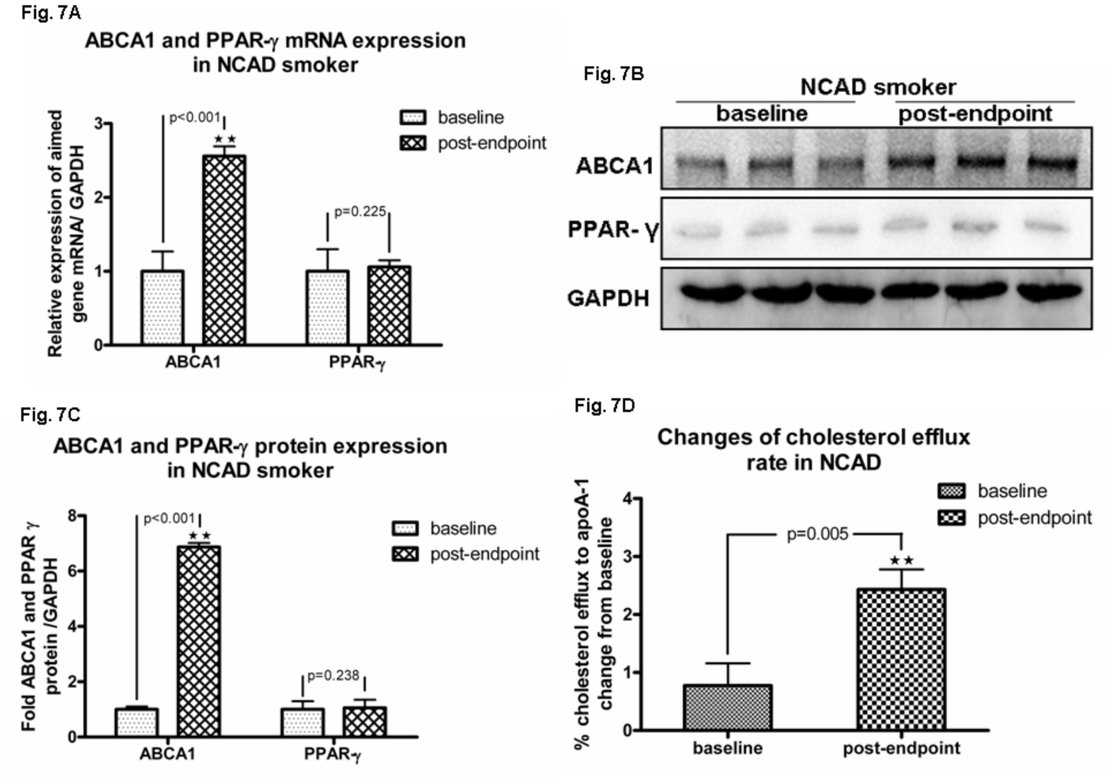

Fig. 7A
ABCA1 and PPAR-γ mRNA expression in NCAD smoker
Relative expression of aimed gene mRNA/ GAPDH
baseline
post-endpoint
p<0.001
p=0.225
ABCA1
PPAR-γ
Fig. 7B
NCAD smoker
baseline
post-endpoint
ABCA1
PPAR- γ
GAPDH
Fig. 7C
ABCA1 and PPAR-γ protein expression in NCAD smoker
Fold ABCA1 and PPAR γ protein /GAPDH
baseline
post-endpoint
p<0.001
p=0.238
ABCA1
PPAR-γ
Fig. 7D
Changes of cholesterol efflux rate in NCAD
% cholesterol efflux to apoA-1 change from baseline
baseline
post-endpoint
p=0.005
baseline
post-endpoint
